# Exploring the Involvement of the Amyloid Precursor Protein A673T Mutation against Amyloid Pathology and Alzheimer’s Disease in Relation to Therapeutic Editing Tools

**DOI:** 10.3390/pharmaceutics14061270

**Published:** 2022-06-15

**Authors:** Gabriela Dumitrita Stanciu, Daniela Carmen Ababei, Razvan Nicolae Rusu, Veronica Bild, Bogdan-Ionel Tamba

**Affiliations:** 1 Advanced Research and Development Center for Experimental Medicine (CEMEX), Grigore T. Popa University of Medicine and Pharmacy, 16 Universitatii Street, 700115 Iasi, Romania; gabriela-dumitrita.s@umfiasi.ro (G.D.S.); bogdan.tamba@umfiasi.ro (B.-I.T.); 2 Pharmacodynamics and Clinical Pharmacy Department, Grigore T. Popa University of Medicine and Pharmacy, 16 Universitatii Street, 700115 Iasi, Romania; razvan.nicolae.rusu@gmail.com (R.N.R.); veronica.bild@gmail.com (V.B.); 3 Department of Pharmacology, Clinical Pharmacology and Algesiology, Grigore T. Popa University of Medicine and Pharmacy, 16 Universitatii Street, 700115 Iasi, Romania

**Keywords:** Alzheimer’s disease, amyloid precursor protein, A673T mutation, therapeutic editing tools, CRISPR/Cas9, cognitive decline, rs63750847

## Abstract

Alzheimer’s disease (AD) is biologically defined as a complex neurodegenerative condition with a multilayered nature that leads to a progressive decline in cognitive function and irreversible neuronal loss. It is one of the primary diseases among elderly individuals. With an increasing incidence and a high failure rate for pharmaceutical options that are merely symptom-targeting and supportive with many side effects, there is an urgent need for alternative strategies. Despite extensive knowledge on the molecular basis of AD, progress concerning effective disease-modifying therapies has proven to be a challenge. The ability of the CRISPR–Cas9 gene editing system to help identify target molecules or to generate new preclinical disease models could shed light on the pathogenesis of AD and provide promising therapeutic possibilities. Here, we sought to highlight the current understanding of the involvement of the A673T mutation in amyloid pathology, focusing on its roles in protective mechanisms against AD, in relation to the recent status of available therapeutic editing tools.

## 1. Introduction

Alzheimer’s disease (AD), a well-known condition associated with old age, is a brain disorder of a multifactorial nature, which leads to cerebral shrinkage (atrophy) and the death of brain cells [1,2,3]. One of the key elements of AD is the accumulation of amyloid-β (Aβ) protein from amyloid precursor protein (APP) through sequential proteolytic cleavage of β- and γ-secretases. Accumulation of Aβ into extracellular senile plaques and hyperphosphorylated tau (p-tau) intracellularly aggregated as neurofibrillary tangles (NFTs) seem to be the most significant neuropathological hallmarks of this condition [4,5,6]. Despite important studies, the etiopathogenesis influenced by epigenetic and genetic variants [7,8], and the mechanisms of both synaptic dysfunction and cognitive decline, are not completely known [9,10,11,12]. Genetic factors refer to aspects such as mutations and polymorphisms which affect susceptibility. Well established mutations associated with the disease are on chromosomes 1, 14 and 21, though at least 24 other genes are also associated [13]. It is considered that genetic factors comprise about one third of risk factors for dementia [14,15,16,17]. Non-genetic factors include depression, hypertension, stroke, diabetes, hypercholesterolemia, obesity, smoking, head trauma and environmental factors, such as exposure to aluminum, copper, zinc, lead, iron, pesticides, solvents, electromagnetic field, air pollution and noise [18,19,20,21,22,23,24,25].

The current therapeutic options offer only symptomatic relief without slowing the disease’s progression, and are accompanied by many side effects [26,27,28,29]. The concept “one molecule–one target–one disease” fails to provide a comprehensive solution for AD therapy. Often, the development of effective therapies encounters difficulties that are based on a multitude of factors, such as gaps in knowledge about the biological pathways and precise molecular alterations [30,31,32]; and the slow recruitment of satisfactory numbers of participants and sufficiently diverse subjects for clinical trials, which is associated with the relatively long time required to detect whether an investigational therapy impacts the course of the disease [33,34,35,36,37]. Progress towards possible therapeutic targets has proven challenging, despite substantial advances regarding the molecular pathogenesis of AD, as a series of clinical trials have failed to meet efficacy standards for biogenesis, toxicity and Aβ production. The first new Alzheimer’s drug in 20 years, aducanumab, was approved for use in humans by the U.S. Food and Drug Administration (FDA) in June 2021—on the condition of further successful trials; representing “a hugely significant milestone” in the search for AD treatments. The drug, an antibody that targets Aβ, could reduce the number of Aβ plaques present in the brain and has the potential to slow down the cognitive deterioration typical of AD [38].

The genetic influence on AD is strong, and genomic data may provide not only insights into the molecular mechanisms underlying the pathogenesis of the disease, but also a perspective on AD prevention and therapy [39,40]. The mutation known as A673T has been shown to decrease the production of Aβ [41]. People carrying this variant present negligible cerebral amyloid deposition, even at the age of 95 [42]. This mutation reduces aggregation of Aβ and the BACE1 cleavage in APP by 40%. It is related to longevity, given that the carriers of A673T are 50% more likely to reach the age of 85 when compared to controls [43]. Confirming this statement, Kero et al. [44] identified a 104.8-year-old A673T mutation carrier, who later died with minor Aβ pathology and a score of zero on the Consortium to Establish a Registry for Alzheimer’s Disease (CERAD) scale. One of the newly developed and most effective pieces of gene editing technology, clustered regularly interspaced short palindromic repeats (CRISPR), gained attention for possible benefits in the fields of basic research and disease therapeutics. This extremely powerful tool can be used as an approach to targeted therapy, to construct better models that mimic human diseases, to uncover novel biological mechanisms or to help screen for pathogenic/protective genes. Moreover, it has proven to be promising for other neurodegenerative conditions, such as Huntington’s and Parkinson’s diseases [45,46,47]. In this evolving landscape, our approach aimed to highlight a cross-disciplinary state-of-art update of the translational literature on the involvement of the A673T mutation in amyloid pathology, focusing on its roles in protective mechanisms against AD, in relation to the recent status of available therapeutic editing tools.

## 2. Amyloid Precursor Protein A673T—Prevalence and Evidence behind Both Biological and Environmental Protection in Alzheimer’s Disease

AD is frequently divided into two categories: early-onset AD (EOAD, 1–5% of all cases of AD and onset before the age of 65) and late-onset AD (LOAD, representing the majority of AD cases, found in people over the age of 65) [48]. For both EOAD and LOAD, there are sporadic and familial forms [49]. The familial forms are most often related to autosomal dominant mutations in genes such as *APP* (amyloid precursor protein), *PSEN1* (presenilin 1) or *PSEN2* (presenilin 2); sporadic ones are suggested to be polygenic, with a more complex etiology [48,50].

**Figure 1:**
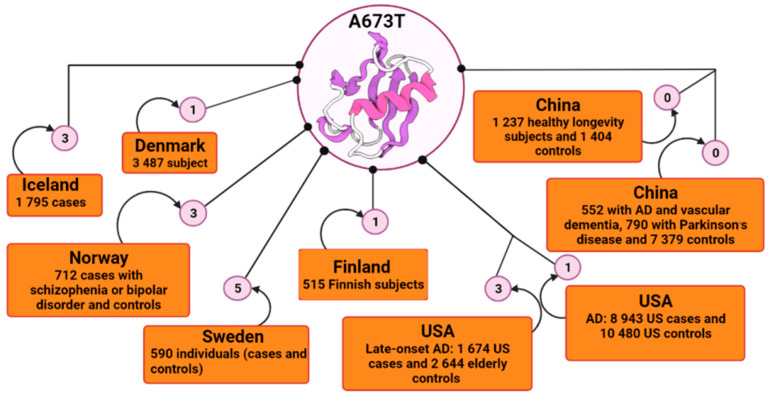
Schematic representation of the incidence of A673T mutation by country using BioRender. In the Icelandic population, the frequency of the A673T mutation was reported to be 0.13% in AD cases and up to about 0.70% in controls. In other large-scale studies, frequencies were reported to be 0.51% for Finnish subjects with AD, 0.42% for Swedish, 0.21% for Norwegian, 0.014% Danish and 0.011% for American; and 0.018% for cognitively normal controls. The A673T mutation appeared to be absent in a screen of 8721 Asian individuals, and in 2641 healthy longevity Chinese subjects. AD, Alzheimer’s disease; USA, United States of America.

*APP*, situated on chromosome 21, is a gene that encodes APP. There are currently more than 60 coding mutations associated with AD in the *APP* gene [51,52,53]; and over 30 of them are pathogenic and increase the risk of autosomal dominant AD, promoting the generation and oligomerization of Aβ, and reducing its clearance [54,55,56,57]. Most of these mutations are close to the proteolytic cleavage sites of β- and γ-secretases that are responsible for Aβ generation [58]. Even though *APP* mutations are most often linked with increased incidence of early-onset familial AD, Jonsson et al. [41] identified a mutation within the *APP* gene in an elderly Icelandic population that has been shown to be protective against AD and is associated with slower cognitive impairment among cognitively normal people. After the first report of this protective mutation by Peacock et al. [59] in a Caucasian individual who died at age 65 but whose cognitive function was good and whose histological analysis did not detect amyloid deposits in the brain, the A673T variant attracted many researchers’ attention. Protection conferred by the A673T mutation was also further supported by a study carried out on people aged 85 years and over who were residents of Vantaa in Finland [44]. Of 601 eligible subjects, 553 underwent clinical examination. Subsequently, 534 subjects had their deoxyribonucleic acid (DNA) sequenced, and 515 subjects were then deemed available for the study. The neuropathological analysis revealed that the signs of β-amyloid pathology were very reduced; there was no amyloid plaque formation [44]. The carrier of the A673T mutation only has light Aβ pathology, age-related neurofilament phosphorylation and cytoplasmatic neurofibrillary tangles [60]. In addition, another study found that plasma Aβ40 and Aβ 42 levels of the *APP* A673T carriers were reduced by an average of 28% compared to their age and *APOE*-matched controls [61]. Consequently, in order to identify the occurrence of the protective A673T mutation, many states begun to conduct extensive sample studies (Figure 1).

In Northern Europe, the A673T variant was also registered among the Norwegian, Danish and Swedish people with a relatively high prevalence [44,62]. The Danish study included participants from three cohorts, elderly people born in 1905, 1910 and 1915; and a younger population of unrelated twins (45–55 years old). Of the total participants, 1651 gave blood samples. The middle-aged Danish twins were assessed physically and cognitively by a battery of tests, none of which showed signs of dementia. Of the blood samples processed, in subjects aged 92–93 years, the A673T variant was not found and in the middle-aged and unrelated group; only 1 out of 744 individuals was found to have the A673T variant present [62].

A study conducted among North American Whites (US Whites) with the purpose of determining the frequency of this variant had 4318 subjects, divided into two cohorts: 1674 late-onset AD cases and 2644 elderly control subjects [63]. All genotyped samples showed the absence of the A673T variant except the positive control, which was entered for verification [63]. However, another study also carried out on the US population showed that this A673T variant is a rare mutation and may be confined to certain races/ethnicities [64]. The aim of the study was to determine the frequency of this mutation in the US population based on genotyping when only three Caucasian individuals were found to be heterozygous for A673T, two of whom were cognitively healthy at ages 77 and 82, respectively. The third carrier developed AD with onset at 89 years of age and was of Russian origin. Three individuals heterozygous for A673T from the Swedish cohort, used as controls, were identified in this study [64].

In order to further explore whether the A673T variant contributes to the Asian population accurately, the researchers genotyped the mutation in two studies [65,66]. One of them recruited 8721 subjects, 552 of whom had AD and vascular dementia, and another study included 2641 subjects, of whom 1237 were long-lived people; in both studies, the population was predominantly ethnically Chinese [65]. Genotyping tests showed that in each of the two situations, no individual carried the A673T mutation in APP, its complete absence suggesting that it may be an ethnically specific variant [66].

It might be considered that the scientific evidence from human A673T screening associated with further preclinical evaluation could be a useful perspective from which to contextualize conclusions regarding the range of Aβ reductions currently sought in clinical trials.

## 3. The Mechanisms of A673T Protection against Amyloid Pathology

It is already well known that missense mutations or duplications of the *APP* gene encode a membrane glycoprotein present in three different isoforms: APP751, APP770 and APP695, the last being the main isoform determined in the brain [67,68,69]. In the normal state, APP cleavage is carried out by α-secretase with the generation of soluble APPα (sAPPα) and a C38 carboxy-terminal fragment. The presence of sAPPα is accompanied by normal synaptic signaling that determines synaptic plasticity, memory and learning, emotional behavior and neuronal survival [70,71,72]. In the pathological state, sequential cleavage of APP is carried out by the β-secretase called BACE1 and γ-secretase, with the release of an extracellular fragment called Aβ40-42, a neurotoxic fragment that frequently aggregates with oligomerization of Aβ40-42 and amyloid plaque formation [70,73,74,75]. The formation of amyloid plaques leads to the occurrence of negative effects: blockage of ion channels and impaired calcium homeostasis, leading to mitochondrial oxidative stress, impaired energy metabolism with abnormal glucose regulation and finally, nerve cell death [76,77].

**Figure 2:**
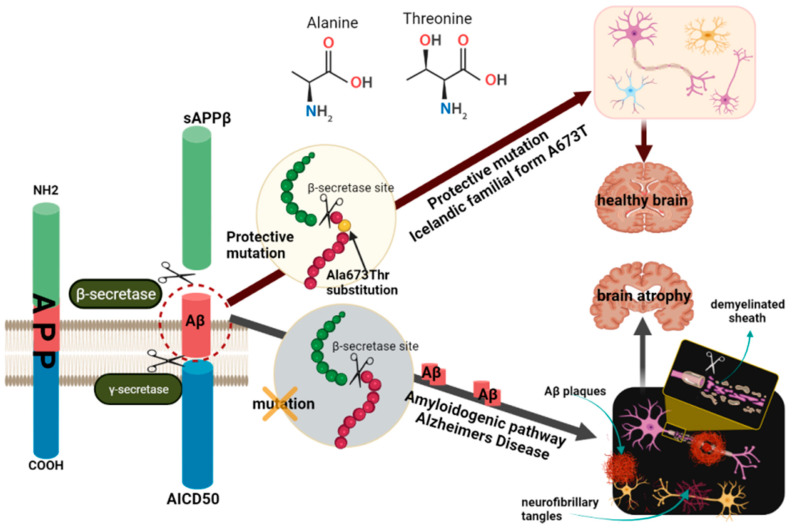
Schematic representation of the molecular mechanisms of A673T protection against amyloid pathology. One of the pathways for APP processing is the formation of toxic amyloidogenic peptides, which accumulate and form amyloid plaques. Two cleavages are required for the release of Aβ from the APP molecule, one in the extracellular domain by β-secretase and another in the transmembrane region by γ-secretase. APP cleavage by β-secretase generates a fragment called β-APP and another smaller fragment that is embedded in the membrane until further cleavage in the presence of γ-secretase. The A673T protective mutation in the APP gene, located near β-secretase, encodes an alanine to threonine substitution. This mutation, also known as A2T, inhibits proteolytic cleavage at the cleavage site of APP by β-secretase. The absence of this mutation leads to cleavage in the presence of γ-secretase, leading to the release of Aβ peptides, especially Aβ42, and the formation of amyloid plaques. Neurofibrillary tangles also form, which together with β-amyloid plaques, lead to impaired synaptic transmission and ultimately to neuronal death. In the case of carriers of the A673T mutation, cleavage by γ-secretase with the formation of the Aβ42 peptide is thus avoided, the carriers being protected from developing AD. The figure was prepared with BioRender. AD, Alzheimer’s disease; APP, amyloid precursor protein; Aβ, amyloid-beta peptide.

A673T mutation, also known as A2T (rs63750847), is an alanine to threonine substitution at amino acid 673 of APP, protective not only against AD development but also cognitive decline in the elderly in general [41]. This substitution is adjacent to the β-secretase cleavage recognition site (Figure 2) and lies in position 2 of the Aβ1-xx peptide cleavage product (A2T). These data suggest that the 673 amino acid position could be critical for the selection of the BACE1 cleavage site during APP processing [33,78,79].

*Aβ generation and aggregation*—A673T mutation can lead to a decreased propensity to generate Aβ by altering the related secretase and metabolites, a process called the amyloidogenetic pathway. Mechanistically, it has been shown that A673T can reduce APP cleavage by BACE1, followed by the formation of an N-terminal APPc89 fragment, contributing to the decrease in APPc99/Aβ [49,80]. Genetic data from a study that replaced alanine with tyrosine highlighted a decrease in APP cleavage by β-secretase and Aβ peptides, suggesting that A673T substitution near the proteolytic site of BACE1 leads to impaired APP cleavage, and may decrease the amyloid generation and accumulation via effects on both APP and Aβ [41]. To confirm these observations, the study was extended in vitro. The A673T mutation in APP was overexpressed in HEK293 cells, a mutation that produced approximately 40% less Aβ40 and Aβ42 than wild-type (WT) APP [41]. In another study, it was shown that carriers of the A673T variant of APP had 28% lower plasma levels of Aβ40 and Aβ42 [61]. Additionally, products such as sAPPβ and β-CTF, resulting from APP cleavage under the action of β-secretase, were likewise reduced. This effect was subsequently confirmed in primary mouse neurons expressing human APP (isoform 695) with A673T [42,81] and in isogenic human induced pluripotent stem cell-derived neurons [42]. Site-directed mutagenesis was used to introduce either the A2T mutation (A673T, Icelandic mutation) or the A2V mutation (A673V, Italian variant; mutation at amino acid position 673 in exon 16, reported first in an Italian family [82]) into the 695 amino acid isoform of human APP. It was found that the A673T mutant significantly reduced β-scission, being considered the protective mutant, and the A673V mutant, due to increased β-scission products, is the disease-causing mutation. Combined in a 1:9 Aβ42/Aβ40 ratio to mimic their heterogeneity in cerebrospinal fluid, Aβ40 containing the A673T maintained a trend toward slowed aggregation kinetics. So far, it is not clear whether this diminished aggregation is primarily due to effects on Aβ40, Aβ42 or both [42,81]. Using steady-state kinetics, the effect of the A673T mutation on APP processing by BACE was also investigated. The results showed that peptides containing the Swedish mutation exhibited the highest peak velocity, and the A673T mutation substrate had a peak velocity value similar to the wild type. The interaction between BACE and the T673 hydroxyl group could be one of the causes affecting the free binding energy of the mutant, suggesting that the mutation affects the catalytic turnover of APP rather than APP affinity for BACE1 [83].

The role of the A673T mutation in N2a cells expressing human APP and containing either the A673T mutation or the A673V mutation together with WT human APP and human APP containing the Swedish mutation (K670N/M671L) was investigated [81]. For each variant, the levels of APP were the same, except for the Swedish mutation, which is sensitive to β-site cleavage. Analysis of alternative cleavage by BACE1 of human APP containing the A673T and A673V mutations indicates they may change the cleavage site selection of BACE1 from β-site to β’-site, providing a cleavage product (11–40) that is significantly more abundant in cells expressing human APP A673T; and conversely, in cells expressing human APP A673V, the production of Aβ (11–40) significantly decreased. These results suggest that this mutation makes APP a less favorable substrate for β-secretase, leading to lower Aβ production and less predisposition to aggregation [80,81,84,85].

Starting from previous research showing that the A673 mutation would have a neuroprotective effect, Zhang et al. [86] examined the role of A673T-substituted Aβ peptides (Aβ40A673T) in mitochondrial axonal trafficking. Cortical neurons were treated with Aβ40 A673T at different concentrations. Aβ40A673T inhibited mitochondrial motility in the retrograde direction to 5%, whereas mitochondrial inhibition with Aβ40 was about 50%. The results of the study suggest that axonal trafficking inhibition was very strong for Aβ peptides with high aggregation potential [86].

*Cellular toxicity of Aβ and microglial uptake*

The cytotoxicity of the Aβ peptide does not appear to be dramatically altered by mutations. The neuronal toxicity associated with mutants Aβ40 and Aβ42 at a range of concentrations was comparable to that of the wild-type Aβ peptide [42]. Neurons expressing the A673T variant were resistant to transforming growth factor β2-induced cell death, but those expressing wild-type APP were not. These findings sustain the idea that the AD-protective mutation of APP decreases the incidence of AD by attenuating the APP-mediated intracellular death signal [87]. Using ion mobility-mass spectrometry, Zheng et al. [88] revealed that Aβ42 with the A673T variant forms dimers, tetramers and hexamers. In contrast, no substantial effects on Aβ40 aggregates were observed, and dodecamer formation was inhibited. Limegrover et al. [89] examined the rate of formation, quantity and intensity of soluble oligomers obtained from synthetic protein containing the Icelandic A673T mutation. Cells taken from the hippocampi and cortical areas of rat embryos on the 18th day of pregnancy were used, and neuronal cultures were treated with various concentrations of the oligomeric protein to examine whether there are differences between A673T and wild-type Aβ42 in relation to binding to neuronal cultures. The results obtained suggest that mutant protein oligomers, compared to wild type oligomers, have a lower affinity for synaptic binding sites: the A673T mutant Aβ42 protein formed approximately 50% fewer Aβ oligomers by weight [89]. Microglial uptake is considered to be a significant clearance mechanism for cerebral soluble Aβ, and the mutant Aβ42 peptides correlated with their aggregation level [41]. Although this mutation is infrequent in non-Nordic countries, the results of the above-mentioned studies strongly support the amyloid hypothesis [44,62,63,64,65,66,90], and the importance of exploring new approaches in order to improve our understanding of the underlying mechanisms and processes affecting the biophysical properties of Aβ peptides [41,42,44,62,63,64,65,66,90]. Reducing Aβ production by approximately 20–40% via A673T mutation can be considered to be protective factor prior to the onset of amyloid generation and accumulation, and also tolerated so as to not impair the native functions of Aβ and APP processing [41,42]. Consequently, these effects could together have an additive or even synergistic impact on the risk of developing AD.

## 4. Looking into the Icelandic Mutation’s Involvement in Alzheimer’s Disease with Gene and Base Editing Tools

**Figure 3:**
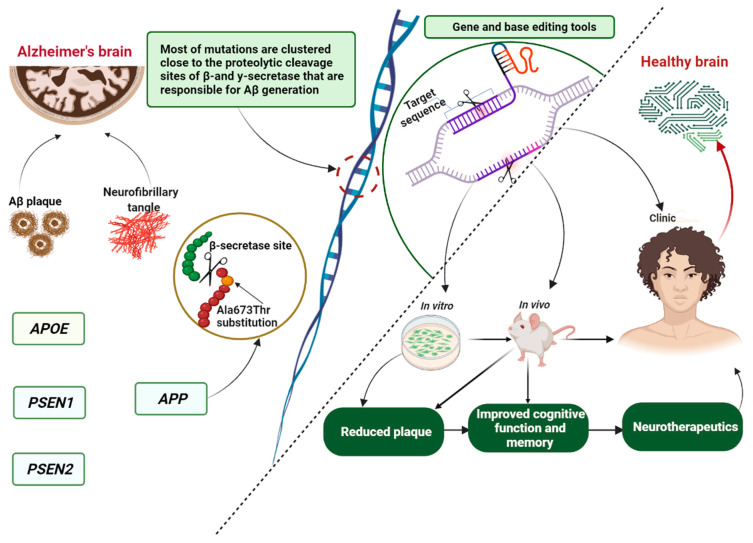
Schematic representation of the involvement of the Icelandic mutation in Alzheimer’s disease regarding genome editing tools. The figure was prepared with BioRender. APP, amyloid precursor protein; Aβ, amyloid-beta peptide; PSEN1, presenilin 1; PSEN2, presenilin 2; APOE, apolipoprotein E.

Repeated failure in clinical trials has challenged our understanding of this multifactorial disease and its irreversibility, leading to recent studies concentrating on advancing our knowledge of the underlying mechanisms of AD progression to find druggable and genomic targets for more effective therapeutic solutions. The role of genetics in disease treatment has generated powerful genome research tools that hold great promise for finding therapeutic candidates or genes that could be therapeutics themselves. At present, the core genome editing technologies mainly include: clustered regulatory interspaced short palindromic repeats (CRISPR-associated protein 9, Cas9) [91], zinc finger nucleases (ZFNs) [92], transcription activator-like effector nuclease (TALENs) [93] and homing meganucleases or endonucleases [94,95,96]. The CRISPR–Cas9 system is one of the most powerful basic and translational pieces of research technology for correcting, writing or deleting inconsistent genetic signatures [97,98,99]. It uses an RNA binding domain, whereas ZENs, TALENs and meganucleases identify and bond via DNA to create a double-standard break by proteins [46,96,100]. The CRISPR–Cas9 gene editing system has been widely used in genetic engineering in the field of AD (Table 1) for the development of preclinical models, pathological gene screening and therapy (Figure 3) via specific target genes, such as APP, APOE, PSEN1, PSEN2, BACE1, glia maturation factor (GMF) and CD33.

Therapeutic genome editing strategies can be achieved through a series of approaches that include the introduction of protective mutations, the inactivation or correction of harmful mutations, the disruption of viral DNA or the addition of therapeutic transgenes [42].

Transgenic cells or animals and genome editing technology may also be pooled to generate preclinical disease models related to the A673T mutation to clarify aspects of the molecular pathways of AD pathogenesis and progression, especially those involved in the mutant amyloidogenic pathway that affects the sequence APP coding [114,115,116,117,118]. Using the CRISPR–Cas9 system, the A673V variant located near the APP β-secretase cleavage site has been shown to contribute to AD pathology by increasing Aβ production and augmenting its aggregation and toxicity [119]. However, the A673T variant, which is adjacent to the aspartyl protease β-site in APP, offers protection against AD evolution [61]. When the two mutations A673V and A673T were introduced into normal human induced pluripotent stem cells (iPSCs) by TALENs technology, these cells differentiated and formed cortical neurons, exhibiting different levels of AD-associated biomarkers [120]. Guyon and collaborators [121] have focused on developing a base editing strategy to insert the A673 mutation into cultured cells using mixed SH-Sy5Y cell lines containing the APP WT gene or the APP gene with the V717I mutation (London mutation) to prevent the development of AD in vitro. Both lines, following treatment with the basic editors, showed reductions in Aβ peptides of 23% and 26%, and Aβ42 peptides of 6.7% and 31.8% [121]. CRISPR–Cas9-mediated genome editing tools have also been used by Tambini et al. [122] for studying APP processing and human Aβ levels in rats carrying either A673T (protective) or Swedish APP pathogenic mutations. The results revealed that the A673T mutation, through a reduction in APP affinity for BACE1, has as a secondary effect: a decrease in Aβ production. Nevertheless, the direct consequences of this mutation are diminution of sAPPβ and βCTF production, the metabolites produced by β-cleavage of APP [121]. Using CRISPR prime editing, a novel and more versatile base editing technique, to insert the A673T mutation into HEK293T cells, Trembaly et al. [122] demonstrated that repeated therapy increased the mutation rate of up to 49.2% with the prime editing technique and up to 68.9% with a simultaneous protospacer adjacent motif. These data raise the possibility that with an optimal delivery system, the A673T variant may be inserted directly in patient’s neurons to prevent hereditary and eventually sporadic AD. Thus, more studies are undoubtedly needed to further refine the link between A673T mutation and its role in disease etiopathogenesis. Overall, genetic findings and gene editing technologies can thus help to better define the hypothetical therapeutic index for the modulation of any human gene while also pointing toward possible off target effects.

## 5. Concluding Remarks

The evidence gathered has highlighted important contributions of genome editing systems to explore safer therapeutic strategies for AD, among which, the CRISPR–Cas9 system has been particularly effective by directly deriving multifunctional tools or interfering with target genes. In the coming years, CRISPR screening, combined with existing data on the genetic and epigenetic AD characteristics of AD, will be capable of identifying interindividual biological differences, synthetic lethal genome interactions or preclinical disease models and facilitate the detection of innovative drug targets. Despite the fact that the off-target effects with the use of these tools still require further optimization and advanced genome editing platforms. Detailed carriers have improved safety, specificity and efficiency during the delivery process, bringing these technologies closer to the clinic. In this conceptual framework, given the recent knowledge that the A673T mutation delays/protects against what is known as normal aging-dependent cognitive decline along with deeper exploration of these editing tools, it is reasonable to believe that genome editing technology has the potential to ultimately elucidate the biological mechanisms behind AD development and progression, which will be conducive to novel therapies.

## Figures and Tables

**Table 1:** Alzheimer’s disease therapeutics using the CRISPR–Cas9 system.

Targeted Genes	Experimental Model	Findings
Amyloid precursor protein (APP)	Tg2576 mice [101]	APP and Aβ reduction
	cell and animal models [102]	β-cleavage and Aβ production attenuation
	human induced pluripotent stem cells [103]	disease model
	cell line [104]	a model of γ-secretase substrate recognition and Notch receptors
3′-UTR APP	C57BL/6 mice [105]	APP and Aβ reduction
Presenilin 1 (PSEN1)	human induced pluripotent stem cells [103]	disease models generated by CRISPR
	SH-SY5Y neuroblastoma cells [106]	identification of homozygous and heterozygous mutations
Presenilin 2 (PSEN2)	human basal forebrain cholinergic neurons [107]	reduced Aβ42/40 ratio
	human basal forebrain cholinergic neurons [108]	normalization of Aβ levels
Beta-secretase 1 (BACE1)	5 × FAD Alzheimer’s mouse model [109]	reduction of APP, Aβ and cognitive impairment
BACE1 and tyrosine hydroxylase (Th)	cell line [110]	Reduction of BACE1, Aβ production and Th
Apolipoprotein E (APOE)	induced pluripotent stem cells [111]	reduction of Aβ deposition and hyper-phosphorylation of tau protein, increased turning of APOE4 to APOE3
	human and murine cell lines [112]	permanent correction of ~15–75% of total cellular DNA with minimal indel formation
γ-secretase activating protein (GSAP)	HEK-APP cell line [113]	reduces γ-secretase activity for Aβ production, but not for Notch1 cleavage

APP, amyloid precursor protein; Aβ, amyloid β; PSEN1, presenilin 1; PSEN2, presenilin 2; CRISPR–Cas9 system, clustered regulatory interspaced short palindromic repeats-associated protein 9; BACE1, beta-secretase 1; APOE, apolipoprotein E; DNA, deoxyribonucleic acid; GSAP, γ-secretase activating protein; Th, tyrosine hydroxylase.

## Data Availability

No new data were created or analyzed in this study. Data sharing is not applicable to this article.
